# Circulating microRNA miR-137 as a stable biomarker for methamphetamine abstinence

**DOI:** 10.1007/s00213-022-06074-z

**Published:** 2022-02-09

**Authors:** Baeksun Kim, Sung Hyun Tag, Yong Sik Kim, Sung Nam Cho, Heh-In Im

**Affiliations:** 1 Convergence Research Center for Diagnosis, Treatment and Care System of Dementia (DTC), Korea Institute of Science and Technology (KIST), Seoul, 02792 Republic of Korea; 2 Division of Bio-Medical Science & Technology, KIST School, Korea University of Science and Technology (UST), Seoul, 02792 Republic of Korea; 3 Department of Pharmacology, Seoul National University College of Medicine, Seoul, 03080 Republic of Korea; 4 Addiction Brain Center, Gangnam Eulji Hospital, Seoul, 06047 Republic of Korea; 5 Center for Neuroscience, KIST, Seoul, 02792 Republic of Korea; 6 Laboratory of Behavioral and Molecular Neuroscience, Brain Science Institute (BSI), Korea Institute of Science and Technology (KIST), Hwarang-ro 14-gil 5, Seongbuk-gu, Seoul, 02792 Republic of Korea

**Keywords:** Methamphetamine, Abstinence, Withdrawal, miRNA, miR-137, Extracellular vesicles, Diagnosis, Biomarker

## Abstract

**Objective:**

Stimulant use instigates abstinence syndrome in humans. miRNAs are a critical component for the pathophysiology of stimulant abstinence. Here we sought to identify a miRNA marker of methamphetamine abstinence in the circulating extracellular vesicles (cEVs).

**Methods:**

miR-137 in the cEVs was quantified by qPCR in thirty-seven patients under methamphetamine abstinence and thirty-five age-matched healthy controls recruited from 2014 to 2016 from the general adult population in a hospital setting, Seoul, South Korea. Diagnostic power was evaluated by area under curve in the receiver-operating characteristics curve and other multiple statistical parameters.

**Results:**

Patients under methamphetamine abstinence exhibited a significant reduction in cEV miR-137. Overall, cEV miR-137 had high potential as a blood-based marker of methamphetamine abstinence. cEV miR-137 retained the diagnostic power irrespective of the duration of methamphetamine abstinence or methamphetamine use. Interestingly, cEV miR-137 interacted with age: Control participants displayed an aging-dependent reduction of cEV miR-137, while methamphetamine-abstinent patients showed an aging-dependent increase in cEV miR-137. Accordingly, cEV miR-137 had variable diagnostic power depending on age, in which cEV miR-137 more effectively discriminated methamphetamine abstinence in the younger population. Duration of methamphetamine use or abstinence, cigarette smoking status, depressive disorder, or antidepressant treatment did not interact with the methamphetamine abstinence-induced reduction of cEV miR-137.

**Conclusion:**

Our data collectively demonstrated that miR-137 in the circulating extracellular vesicles held high potential as a stable and accurate diagnostic marker of methamphetamine abstinence syndrome.

**Supplementary Information:**

The online version contains supplementary material available at 10.1007/s00213-022-06074-z.

## Introduction

Abstinence syndrome is a critical subdivision of substance-induced disorders. In humans, abstinence syndrome develops and persists for years after cessation of prolonged substance use (Cruickshank and Dyer 2009). Abstinence syndrome causes significant impairment in our daily lives, most notably within the social and occupational spheres (American Psychiatric Association 2013). However, the biological diagnosis of substance abstinence syndrome has been hampered by limitations of the current diagnostic method, specifically the limited window of detection and controversial accuracy (Dolan et al. 2004; Fiorentin et al. 2018; Jarvis et al. 2017; Taylor et al. 2017).

Methamphetamine (MA) is a white-colored, odorless, synthetic stimulant derived from substituting the amine group into methyl group in amphetamine. MA is known for its notoriously addictive property, as MA could easily disrupt the blood-brain barrier to open the way into CNS and is slowly metabolized in the brain (Northrop and Yamamoto 2015; Volkow et al. 2010). The consequences of chronic MA use are severe. The people who chronically abused MA can exhibit symptoms including anxiety, mood disturbance, psychosis, decline in mental flexibility, decreased attention, motor impairment, insomnia, aggressive behavior, increased appetite, and more (American Psychiatric Association 2013). These symptoms collectively belong to MA abstinence syndrome, which can last from months to years after a person has quit abusing MA (McGregor et al. 2005).

MA use is marked by aberrant alterations in the expression of brain microRNAs (miRNAs), which are endogenous small non-coding RNAs that play a critical role in the post-transcriptional control of gene repression (Bartel 2004; O'Brien et al. 2018). Interestingly, studies have reported that (1) miRNAs in the circulating extracellular vesicles (cEVs) are prone to modifications by brain diseases (Shah et al. 2018), and (2) can circulate throughout the bloodstream with remarkable stability (Köberle et al. 2013; Zhou et al. 2016). These findings suggest that miRNAs within the cEVs may serve as a stable blood-based marker of MA abstinence.

Here we focused on the brain-enriched and evolutionarily conserved miRNA miR-137 in the cEVs. Independent studies have demonstrated that substance use is marked by the dysregulation of miR-137 in the striatum (Cabana-Dominguez et al. 2018; Quinn et al. 2018; Schaefer et al. 2010). In addition, miR-137 is a known psychiatric risk gene (Cheng et al. 2018; Siegert et al. 2015), and its genetic variant has cross-disorder effects among multiple psychiatric disorders (Consortium 2013). Moreover, miR-137 is known to be brain-enriched (Mahmoudi and Cairns 2017; Sempere et al. 2004), and the brain-enriched miRNAs can circulate throughout the bloodstream (Zhou et al. 2016), which indicates that this miR-137 holds the potential to reflect the MA abstinence-induced changes in brain dysfunction.

In this study, we probed the diagnostic potential of miR-137 in the cEVs of human patients under MA abstinence. In addition, we evaluated the interaction between circulating miR-137 and other clinical factors (age, duration of MA use, duration of MA abstinence, smoking status, depressive disorder, antidepressant treatment). To briefly summarize, we found that MA abstinence substantially reduces cEV miR-137 in human patients. In-depth analysis revealed that cEV miR-137 was not influenced by the duration of MA use or abstinence but was influenced by age. The MA abstinence-dependent reduction in cEV miR-137 was maintained in both younger and older participants, but cEV miR-137 exhibited higher diagnostic power for MA abstinence in the younger participants. Lastly, cEV miR-137 did not interact with cigarette smoking, comorbid depression, or antidepressant treatment in the MA-abstinent patients. Collectively, we propose through this preliminary discovery cohort study that cEV miR-137 holds potential as a blood-based diagnostic marker of methamphetamine abstinence in humans.

## Methods

### Study design

The study’s objective was to discover a blood-based biomarker for methamphetamine abstinence. miR-137 was quantified in the serum extracellular vesicles of human patients and age-matched healthy controls. Diagnostic power was derived through appropriate statistical analyses. Sample sizes were determined on the basis of previous human studies. The investigators were blinded to allocation during experiments and outcome assessment.

### Human participant recruitment

The clinical study was approved by the Institutional Review Board of Bugok National Hospital (IRB code No. 5-002) and KIST (IRB code No. 2015-001). Patients enrolled in the study were self-admitted males (self-report) subsequently diagnosed with substance use disorder for methamphetamine (MA) according to the *Diagnostic and Statistical Manual of Mental Disorders, Fifth Edition (DSM-5)*. Patients were under self-admission program to abstain from using MA, in which the patients received a formal diagnosis based on physical/mental symptoms relevant to methamphetamine use/abstinence and psychiatric inpatient treatment if applicable. Age- and gender-matched healthy subjects were set as control. Whole blood samples were collected from the subjects that provided informed consent after fully acknowledging the nature and possible consequences of the study.

### Blood sample preparation

Blood samples from human participants were collected by the pre-operative blood draw. Ten-milliliter serum separator tubes were used to collect 5 ml blood samples, and the samples were immediately subjected to serum isolation followed by circulating extracellular vesicle (cEV) isolation.

### Isolation of circulating extracellular vesicles

ExoQuick (System Biosciences, Mountain View, CA, USA) was used for the isolation of cEVs. An appropriate amount of ExoQuick was mixed with the serum samples following the manufacturer’s protocol. Then the mixture was refrigerated at 4°C for 1 h and centrifuged at 2,000*g* for 15 min. The supernatant was removed by aspiration, and the pelleted fraction (cEV) was resuspended in either DEPC-DW for RNA isolation.

### RNA extraction

Total RNA from brain or EV samples was isolated using RNA STAT-60 (AMS Biotechnology, Abingdon, UK) or TRIzol reagent (Thermo Fisher Scientific, MA, USA) according to the manufacturer’s protocol. RNA yield and quality were determined using NanoDrop 2000 UV-Vis Spectrophotometer (Thermo Fisher Scientific).

### Quantification of mature miRNAs

Fifty nanograms of total RNA from each sample was used for cDNA preparation through reverse transcription. For miRNAs, cDNA was amplified using TaqMan Universal Master Mix II, no UNG (Thermo Fisher Scientific) according to the manufacturer’s protocol. Thereafter, the expression of mature miRNAs was quantified using qPCR with TaqMan MicroRNA Assays (Thermo Fisher Scientific). qPCR reactions were run on CFX connect (Bio-Rad). All reactions were performed in triplicates. The relative abundance of miRNAs was calculated by 2^-ddCt^ method (Livak and Schmittgen 2001). A reference miRNA miR-16 was used as a normalization control for the quantification of miR-137 in the cEVs.

### Statistical analysis

Student’s *t*-test and one-way analysis of variance (ANOVA) followed by Dunnett’s post hoc test were conducted when appropriate. The receiver-operating characteristics curve analyses for the human dataset tested the null hypothesis that the area under curve (AUC) is equal to 0.5. *p*-value was determined from the *z*-test, in which two-tailed normal distribution by a *z*-ratio was defined by *z* = (AUC-0.5)/(standard error). Linear regression was used to question whether the slope is significantly non-zero in the relationship between two variables in XY scatter plots. Above statistical analyses were conducted with Prism v6.0 (GraphPad, CA, USA). For all empirical tests, *p* < 0.05 was considered statistically significant. Significance was denoted as *p < 0.05, **p < 0.01, ***p < 0.001, and ****p < 0.0001 unless noted otherwise. Data were displayed as mean ± standard error of the mean (SEM) unless noted otherwise.

Statistical power and effect size of the human dataset were calculated with G*Power 3 (Heinrich-Heine-University Düsseldorf, Düsseldorf, Germany) (Faul et al. 2007), while diagnostic accuracy, specificity, sensitivity, positive predictive value (PPV), and negative predictive value (NPV) were calculated as described in the previous report (Glas et al. 2003).

## Results

To test whether the psychiatric risk gene miR-137 in the circulating extracellular vesicles (cEVs) could be utilized as a blood-based marker of methamphetamine (MA) abstinence in the clinic, we recruited 37 male patients with a history of MA use and 35 control participants for a preliminary discovery cohort study. The demographics of the recruited participants are reported in Table 1.

**Table 1:** Demographics for the discovery cohort study. Younger, age <50 years old; older, age ≥50 years old, *NS* nonsmoker, *S* smoker, *AbD* methamphetamine (MA) abstinence duration, *ND* nondepressed, *D* depressed, *NAD* not on antidepressant, *AD* on antidepressant, *UseD* duration of MA use. Values are expressed as mean ± standard deviation for Age, MA use, MA abstinence, and # of cigarettes smoked per day.

**Demographics**	Control					MA abstinence												
	Total	Younger	Older	NS	S	Total	Younger	Older	NS	S	AbD ≤3mo	AbD >3mo	ND	D	NAD	AD	UseD ≥20yrs	UseD <20yrs
Sample size	35	19	16	23	12	37	22	15	5	32	20	17	8	29	20	17	22	15
Age (years)	48.5±12.3	38.5±5.4	60.3±5.5	45.9±9.8	53.3±15.3	47.5±7.8	42.5±5.4	54.9±4.0	55.4±6.1	46.3±7.4	46.0±7.1	49.3±8.5	44.5±9.0	48.3±7.4	45.7±8.7	49.7±6.1	51.7±5.0	41.3±6.9
MA use (years)	**-**	**-**	**-**	**-**	**-**	18.0±8.1	14.5±6.7	23.1±7.2	23.2±6.4	17.2±8.1	16.2±8.5	20.1±7.2	14.9±5.7	18.9±8.5	16.5±8.2	19.8±7.7	23.3±4.8	10.3±4.9
AbD (years)	**-**	**-**	**-**	**-**	**-**	1.1±1.5	0.9±1.2	1.5±1.7	1.6±2.1	1.0±1.4	0.1±0.1	2.3±1.4	0.9±1.0	1.2±1.6	1.3±1.8	0.9±1.0	1.3±1.7	0.8±0.9
Depression (%)	**-**	**-**	**-**	**-**	**-**	78.4	72.7	86.7	100	86.5	80.0	76.5	0	100	60	100	86.4	66.7
Smoker (%)	34.3	21.0	50.0	0	100	86.5	95.5	73.3	0	100	90.0	82.3	100	82.8	90	82.4	81.8	93.3
# of cigarettes smoked per day	5.7±9.1	3.3±7.5	8.6±10.1	0	16.8±7.4	15.9±12.2	17.2±11.5	14.0±13.4	0	18.4±11.2	17.9±12.0	13.6±12.5	18.5±11.3	15.2±12.5	18.6±13.2	12.7±10.4	18.2±12.8	12.5±10.8

**Fig. 1:**
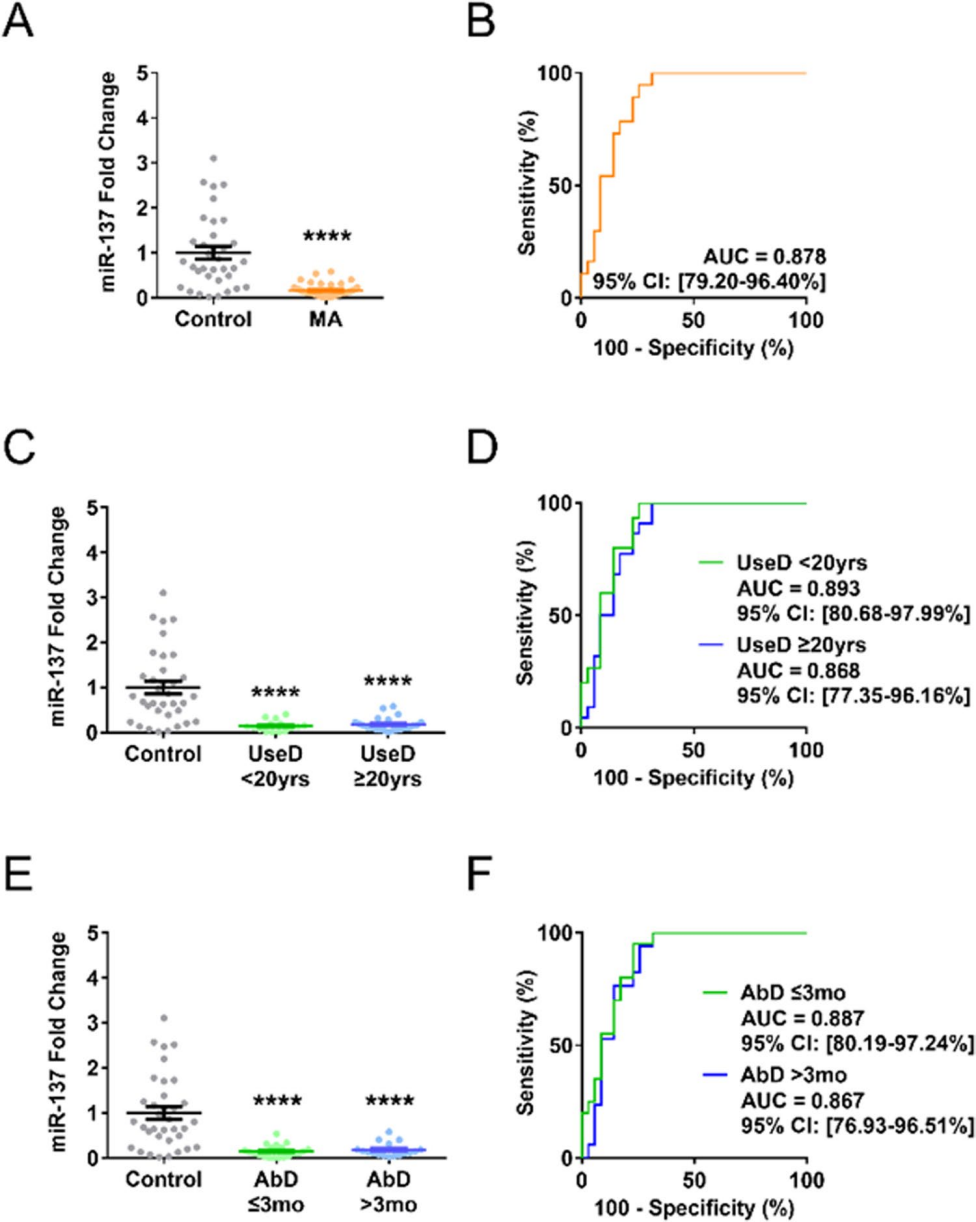
Methamphetamine (MA) abstinence leads to stable, enduring reduction of circulating extracellular vesicle (cEV) miR-137 in humans. **A** Patients under MA abstinence showed a significantly lower expression of cEV miR-137 (*n* = 35 in control, *n* = 37 in MA abstinence) (Student’s *t*-test). **B** Receiver-operating characteristics curve analysis showed that cEV miR-137 had a moderate diagnostic performance for discrimination of MA abstinence (*z*-test). **C** cEV miR-137 was constantly reduced in the MA-abstinent patients irrespective of the duration of MA use (UseD) (*n* = 35, 22, 15 respectively in control, UseD ≥20yrs, and UseD <20yrs) (Dunnett’s post hoc test). **D** In both UseD ≥20yrs and UseD <20yrs, cEV miR-137 showed moderate diagnostic power for MA abstinence (*z*-test). **E** cEV miR-137 was stably decreased in the MA-abstinent patients irrespective of the abstinence duration (AbD) (*n* = 35, 20, 17 respectively, in control, AbD ≤3mo, and AbD >3mo) (Dunnett’s post hoc test). **F** In both AbD ≤3mo and AbD >3mo, cEV miR-137 showed moderate diagnostic power for MA abstinence (*z*-test). 20yrs indicates 20 years. 3mo indicates 3 months. All error bars represent SEM. All experiments were performed once

First, we examined whether the patients undergoing MA abstinence exhibited altered expression of miR-137 in the cEV. qPCR analysis showed that cEV miR-137 was notably reduced in the MA-abstinent patients (Figure 1a). To gauge the diagnostic potential of cEV miR-137 for MA abstinence, we calculated the area under curve (AUC) in the receiver-operating characteristics curve. The AUC of cEV miR-137 in MA abstinence versus control was 0.878 (Figure 1b), with a diagnostic accuracy of 84.7% (Table 2).

**Table 2:** Summary of statistical parameters summarizing the diagnostic potential of circulating extracellular vesicle (cEV) miR-137 for methamphetamine (MA) abstinence. *AUC* area under curve, *CI* confidence interval, *PPV* positive predictive value, *NPV* negative predictive value; younger, age <50 years old; older, age ≥50 years old; *AbD* MA abstinence duration, *NS* nonsmoker, *S* smoker, *ND* nondepressed, *D* depressed, *NAD* not on antidepressant, *AD* on antidepressant, *UseD* duration of methamphetamine use.

**Statistics**	Control vs. MA (Overall)	Control vs. MA Younger)	Control vs. MA (Older)	Control vs. AbD ≤3mo	Control vs. AbD >3mo	Control vs. MA-NS	Control vs. MA-S	Control vs. MA-ND	Control vs. MA-D	Control vs. MA-NAD	Control vs. MA-AD	Control vs. UseD ≥20yrs	Control vs. UseD <20yrs
AUC (%)	87.8	97.8	72.9	88.7	86.7	82.9	88.6	87.5	87.9	86.6	89.2	86.8	89.3
95% CI for AUC (%)	79.2–96.4	93.5–102.2	55.1–90.8	80.2–97.2	76.9–96.5	68.5–97.3	80.0–97.1	77.2–97.8	79.3–96.5	76.8–96.3	80.8–97.7	77.4–96.2	80.7–98.0
Accuracy (%)	84.7	97.5	71.0	83.6	80.8	72.5	85.1	81.4	82.8	83.6	78.9	80.7	82.0
Sensitivity (%)	94.6	100	100	95.0	94.1	100	96.9	100	100	95.0	100	100	100
Specificity (%)	74.3	94.7	43.8	77.1	74.3	68.6	74.3	77.1	68.6	77.1	68.6	68.6	74.3
PPV (%)	79.5	95.7	62.5	70.4	64.0	31.3	77.5	50.0	72.5	70.4	60.7	66.7	62.5
NPV (%)	92.9	100	100	96.4	96.3	100	96.3	100	100	96.4	100	100	100
Achieved power	1.0	1.0	0.9	1.0	1.0	0.7	1.0	1.0	1.0	1.0	1.0	1.0	1.0
Effect size	1.4	2.6	1.3	1.5	1.4	1.2	1.5	1.5	1.4	1.4	1.4	1.4	1.4

Second, we probed whether miR-137 in the cEVs was affected by the duration of MA use. Patients were divided into two groups based on MA use duration (UseD, under and over 20 years; Table 1). We questioned whether the extremely protracted duration of MA use could differentially regulate cEV miR-137. Follow-up analysis showed that the MA abstinence-induced reduction in cEV miR-137 was stable irrespective of the duration of MA use (Figure 1c), and the diagnostic power also remained unchanged by the duration of MA use (Figure 1d; Table 2).

Third, we checked whether miR-137 in the cEVs was stably reduced by MA abstinence irrespective of the duration of MA abstinence. Patients were divided into two groups based on abstinence duration (AbD, under and over 3 months; Table 1). A previous study demonstrated that cue-induced craving for MA elevated until 3 months of abstinence but reduced thereafter (Wang et al. 2013), suggesting that the 3-month time point is a turning point in the course of MA abstinence. Subsequent statistical analysis showed that cEV miR-137 was stably reduced throughout the duration of MA abstinence (Figure 1e), and the diagnostic power also remained unchanged by the abstinence duration (Figure 1f; Table 2).

**Fig. 2:**
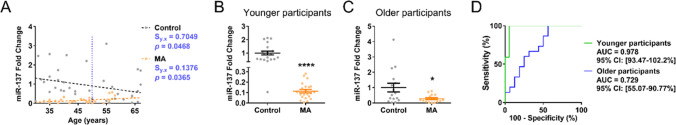
Age interacts with circulating extracellular vesicle (cEV) miR-137 and its diagnostic power in methamphetamine (MA) abstinence. **A** The fold change of cEV miR-137 as a function of age revealed that cEV miR-137 was significantly increasing in response to aging in the MA-abstinent patient group while decreasing in response to aging in the healthy control group (linear regression). Blue dotted line indicates the separation criterion (50 years old). **B** In the younger participants (<50 years old) (*n* = 19 in control, 22 in MA), cEV miR-137 was significantly reduced by MA abstinence (Student’s *t*-test). **C** In older participants ≥50 years old) (*n* = 16 in control, 15 in MA), cEV miR-137 was significantly reduced by MA abstinence (Student’s *t*-test). **D** Receiver-operating characteristics curve analysis showed that cEV miR-137 had higher diagnostic power for MA abstinence in the younger participants but had lower diagnostic power in older participants (*z*-test). All error bars represent SEM. All experiments were performed once

Age affects miRNA contents in the extracellular vesicles (Dluzen et al. 2017). Therefore, we investigated whether aging alters the level of cEV miR-137. Interestingly, aging-dependent changes were apparent in cEV miR-137 in both the MA-abstinent patients and healthy participants. Linear regression analysis showed that while the MA-abstinent patient group exhibited an aging-dependent increase in cEV miR-137 towards the control level, the healthy participant group exhibited an aging-dependent decline of cEV miR-137 (Figure 2a).

We suspected that the diagnostic power of cEV miR-137 for MA abstinence could be aging-dependently modulated. To explore this possibility, we divided the participants based on overall median age (under and over 50 years old, described as younger and older participants, respectively) (Table 1). Older patients typically experience worse symptoms of MA abstinence (McGregor et al. 2005), which might be correlated with the finding that fewer people aged over 50 years old reported past-year MA use compared to the adults aged under 50 years old (Jones et al. 2020).

Subsequent analysis of diagnostic power showed that miR-137 in the cEVs was significantly reduced by MA abstinence regardless of aging, but the fold reduction was larger in the younger participants compared to the older participants (diminished to 11.28% and 27.86% of control in younger and older participants, respectively) (Figure 2b, c). More importantly, the diagnostic power of cEV miR-137 for MA abstinence was significantly greater in the younger participants than the older participants (AUC of 0.978 and 0.729, respectively) (Figure 2d), with the diagnostic accuracy reaching 97.5% in younger participants and 71.0% in older participants (Table 2). However, the within-group analysis suggested that the aging-dependent changes in cEV miR-137 were minor, as the power of cEV miR-137 for discriminating young and old within either the healthy control or MA-abstinent participants was low (Figure S1).

To confer validity to the diagnostic potential of cEV miR-137 for MA abstinence, we surveyed the impact of confounding factors frequently associated with MA abstinence: Cigarette smoking status, comorbid depressive disorder, and antidepressant treatment.

**Fig. 3:**
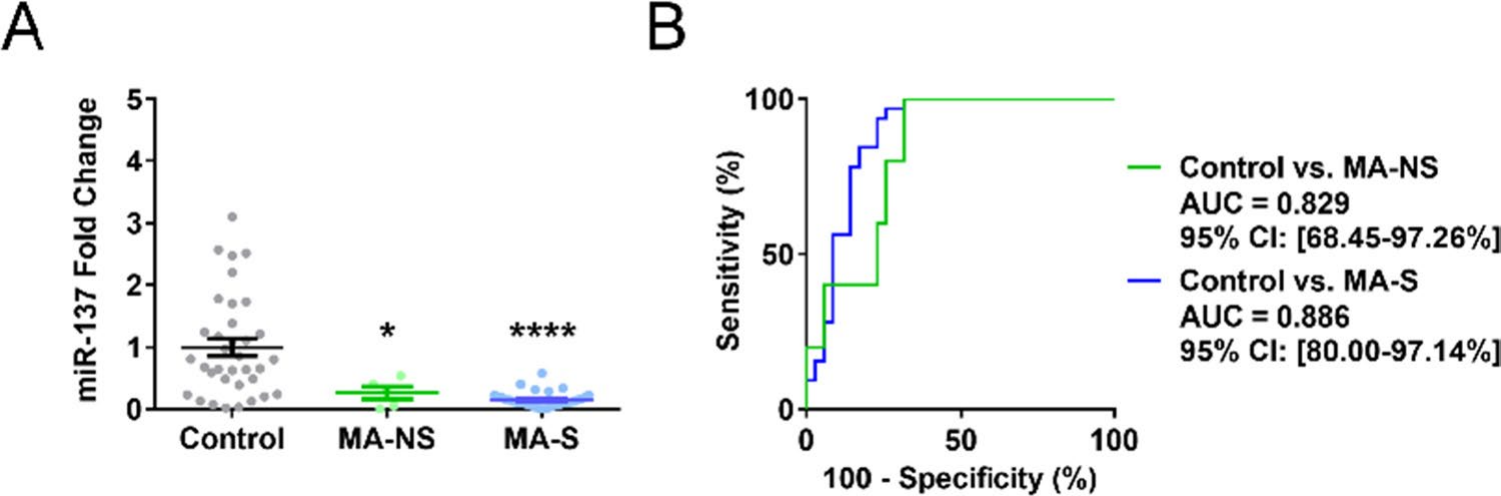
Cigarette smoking does not interact with the diagnostic power of circulating extracellular vesicle (cEV) miR-137 for methamphetamine (MA) abstinence. **A** Cigarette smoking did not significantly affect cEV miR-137 in MA-abstinent patients (*n* = 35, 32, 5, respectively, in control, MA-S, and MA-NS) (Dunnett’s post hoc test). **B** In both MA-NS and MA-S, cEV miR-137 displayed moderate diagnostic power for MA abstinence (*z*-test). MA-NS and MA-S indicate nonsmoking and smoking patient groups, respectively. All error bars represent SEM. All experiments were performed once

First, we examined the potential effect of smoking status on cEV miR-137. We divided the patients based on cigarette smoking status (smokers and nonsmokers, respectively denoted as S and NS). Subsequent analysis demonstrated that cigarette smoking may not interact with the MA abstinence-induced reduction of cEV miR-137 (Figure 3a), and the diagnostic power of cEV miR-137 for MA abstinence was unaffected by cigarette smoking (Figure 3b; Table 2). In addition, cigarette smoking did not alter cEV miR-137 within the healthy control participants and within MA-abstinent patients (Figure S2).

**Fig. 4:**
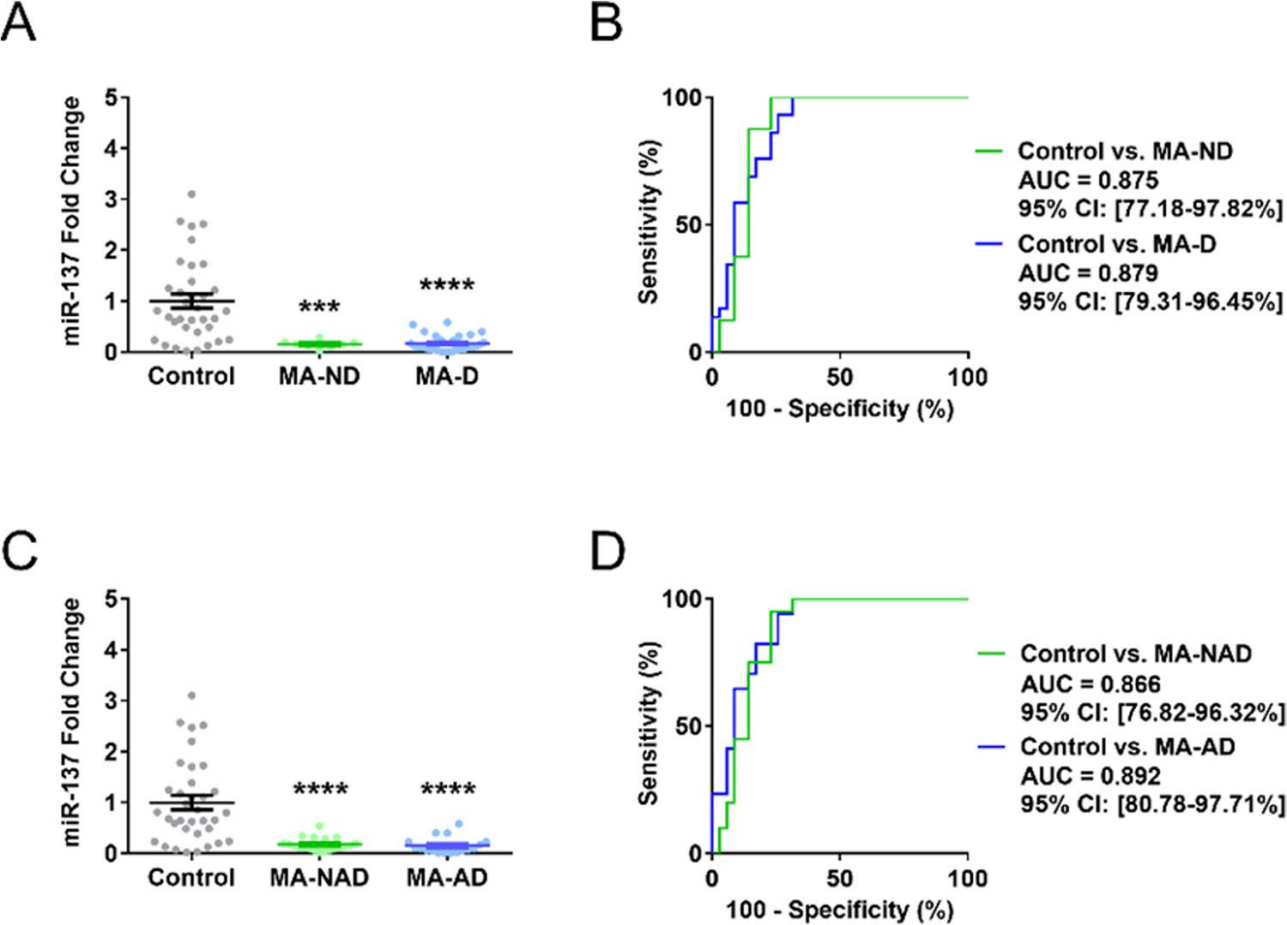
Comorbid depressive disorder or antidepressant medication does not interact with the diagnostic power of circulating extracellular vesicle (cEV) miR-137 for methamphetamine (MA) abstinence. **A** Comorbid depressive disorder did not affect the MA abstinence-induced reduction of cEV miR-137 (*n* = 35, 29, 8, respectively, in control, MA-ND, and MA-D) (Dunnett’s post hoc test). **B** In both MA-ND and MA-D, cEV miR-137 showed moderate diagnostic power for MA abstinence (*z*-test). **C** Antidepressant treatment id not significantly affect the MA abstinence-induced reduction of cEV miR-137 (*n* = 35, 20, 17, respectively, in control, MA-NAD, and MA-AD) (Dunnett’s post hoc test). **D** In both MA-NAD and MA-AD, cEV miR-137 showed moderate diagnostic power for MA abstinence (*z*-test). MA-ND and MA-D indicate nondepressed and depressed patient groups, respectively. MA-NAD and MA-AD indicate patients not on antidepressant or on antidepressant, respectively. All error bars represent SEM. All experiments were performed once.

Next, we assessed the potential impact of depressive disorder or antidepressant treatment on cEV miR-137 in MA-abstinent patients. We divided the patients based on the comorbid depressive disorder or antidepressant use (ND for nondepressed, D for depressed, AD for on antidepressant medication, and NAD for not on antidepressant medication; Table 1). Subsequent analysis showed that cEV miR-137 interacted with neither comorbid depressive disorder nor antidepressant treatment in the MA-abstinent patients (Figure 4a, c), and the discriminative power of cEV miR-137 for MA abstinence was unaffected by depressive disorder or antidepressant (Figure 4b, d; Table 2).

These data demonstrated that the confounding clinical factors associated with MA abstinence (cigarette smoking, depressive disorder, or antidepressant medication) did not influence the diagnostic potential of cEV miR-137 for MA abstinence. However, caution is needed in the interpretation of these results, as other unaccounted or unknown factors could interact with the expression patterns of cEV miR-137

## Discussion

Blood-based markers have gained attention in the last decade due to their considerable merit for in vitro diagnostics (Van den Berg et al. 2020; Weiland et al. 2012). Here we explored the diagnostic potential of miR-137 in the circulating extracellular vesicles for methamphetamine abstinence.

Our data demonstrated that miR-137 in the circulating extracellular vesicles has potential as a blood-based marker of methamphetamine abstinence in humans, with a large window of detection and high stability of diagnostic power. In addition, our data demonstrated that circulating miR-137 weakly interacts with aging in the within-group comparison (Figure S1) but significantly interacts in the between-group comparison so that circulating miR-137 may be more effective at detecting methamphetamine abstinence in the younger population.

Methamphetamine users and past users display an extremely high rate of cigarette smoking (Salo et al. 2011; Weinberger and Sofuoglu 2009). In addition, methamphetamine-abstinent patients are frequently afflicted with depressive disorder (Cruickshank and Dyer 2009; McGregor et al. 2005). However, we found that cigarette smoking, comorbid depression disorder, or antidepressant treatment is unlikely to interact with the diminished miR-137 in the circulating extracellular vesicles of methamphetamine-abstinent patients.

### miR-137 in the circulating extracellular vesicles

Recent studies demonstrated that miR-137 plays a role in drug addiction (Cabana-Dominguez et al. 2018; Quinn et al. 2018), yet the potential function of miR-137 in drug addiction is largely unknown. Previous studies found that miR-137 essentially regulates neuronal development (Mahmoudi and Cairns 2017) and synaptic plasticity (Loohuis et al. 2015; Siegert et al. 2015). These findings suggest that miR-137 could play crucial roles in the aberrant behaviors and neuropathological mechanisms relevant to methamphetamine abstinence as well as addiction.

Furthermore, the multifaceted roles of miR-137 on neuronal functions and dysfunctions suggest that circulating miR-137 could reflect neuronal dysfunction induced by methamphetamine abstinence. However, the functional roles of circulating miR-137 in animals remain unexplored to date. Thus, further studies are warranted to identify the biological/neuronal functions as well as the symptomatic correlate of miR-137 in the circulating extracellular vesicles.

### Comparison of miR-137 with other miRNA markers of methamphetamine-related disorder

Previously, three studies explored the diagnostic potential of circulating miRNAs for methamphetamine use disorder (Gu et al. 2020; Sandau et al. 2020; Zhao et al. 2016). Sandau and colleagues focused on the miRNAs in the plasma extracellular vesicles (EVs), while others focused on serum/plasma miRNAs. Gu et al. and Sandau et al. showed that serum miR-9-3p and plasma EV miR-125b-5p had the highest diagnostic potential for methamphetamine use disorder (AUC = 0.743 and 0.830), respectively. On the other hand, Zhao and colleagues showed that plasma miR-15b was most significantly decreased by methamphetamine use disorder (fold reduced to 47.62% of control), but did not display diagnostic power of miR-15b for methamphetamine use disorder.

In comparison, our study is distinct in that (1) the diagnostic potential of miRNA in the circulating extracellular vesicles for methamphetamine abstinence was unexplored to date and that (2) several different confounding factors were taken into consideration during the investigation of the discriminative power of circulating miR-137 for methamphetamine abstinence.

### Limitations and further study

First, the small sample size in this preliminary discovery cohort study precludes from asserting circulating miR-137 as a diagnostic marker. For instance, the notably small sample size in the MA-NS group impedes us from clearly stating that cigarette smoking does not affect the methamphetamine abstinence-induced reduction of circulating miR-137. At least in principle, we showed that the achieved power for the statistical comparison of control versus MA-NS group is 0.7, which is acceptable according to previous studies (Drouin et al. 2009; Jiang et al. 2004), and all other statistical comparisons resulted in the statistical power of 0.9–1.0 (Table 2). Still, a validation study with a larger, independent cohort must follow in order to establish miR-137 in the circulating extracellular vesicles as a blood-based marker of methamphetamine abstinence.

Second, the potential interaction between age and other comorbid factors (cigarette smoking, depression, etc.) should be taken into consideration. For instance, the MA-NS group was older than the MA-S group, suggesting that age could influence circulating miR-137 in MA-NS and MA-S groups. Here we note that age does not have an overt within-group effect on circulating miR-137. Although linear correlation did exist between circulating miR-137 and age within the group of either control or MA-abstinent patients (Figure 2A), the within-group comparison of circulating miR-137 was not significant between younger control and older control (Figure S1A-B) or younger MA and older MA (Figure S1C-D). These findings suggest that age does not critically influence circulating miR-137 but rather moderately interacts with the effect of methamphetamine abstinence on circulating miR-137. However, the interaction between age and depression disorder or other comorbid conditions should be investigated in-depth in the future.

Third, the potential interaction among the confounding factors (cigarette smoking, depression, antidepressant treatment, etc.) should also be taken into consideration. Here we tried to focus on the interaction between each factor and miR-137 in the circulating extracellular vesicles. If we were to identify the interaction among the confounding factors, the participants would have been divided into much smaller groups. In such case, the number of participants in each group would be substantially reduced so that statistical power becomes smaller and may compromise the statistical validity. We hope that the potential interaction among the confounding factors be investigated in the follow-up study.

Fourth, previous studies have shown that miR-137 can be used as a marker for a number of brain diseases including Alzheimer disease and schizophrenia (Geekiyanage et al. 2012; Ma et al. 2018). Therefore, inclusion of miR-137 in a distinct panel of multiple circulating miRNA biomarkers for methamphetamine abstinence would be effective as with the biomarker panel suggested for Alzheimer disease or depression (Garbett et al. 2015; Leidinger et al. 2013; Ray et al. 2007).

## Conclusion

miR-137 in the circulating extracellular vesicles holds diagnostic potential as a blood-based marker of methamphetamine abstinence. Reduction in circulating miR-137 was stable across >20 years of methamphetamine abstinence, and its diagnostic accuracy reached up to 97.7%, thereby overcoming the major limitations of the conventional biological diagnostics (limited window of detection and questionable accuracy) (Dolan et al. 2004; Fiorentin et al. 2018; Jarvis et al. 2017; Taylor et al. 2017). However, we note that a follow-up validation cohort study is required to establish the clinical utility of miR-137 in the circulating extracellular vesicles as a blood-based marker of methamphetamine abstinence.

## Supplementary Information

Fig. S1 (PNG 935 kb)

High resolution image (TIF 656 kb)

Fig. S2 (PNG 952 kb)

High resolution image (TIF 681 kb)

## Data Availability

Datasets supporting the findings of this paper are available within the paper and can be provided from the corresponding author upon request.
