# Supplementary figures and images for: Circulating microRNA miR-137 as a stable biomarker for methamphetamine abstinence
Source: Psychopharmacology (Berl). 2022 Feb 9;239(3):831–40. doi: 10.1007/s00213-022-06074-z (PMC8891205; doi:10.1007/s00213-022-06074-z)

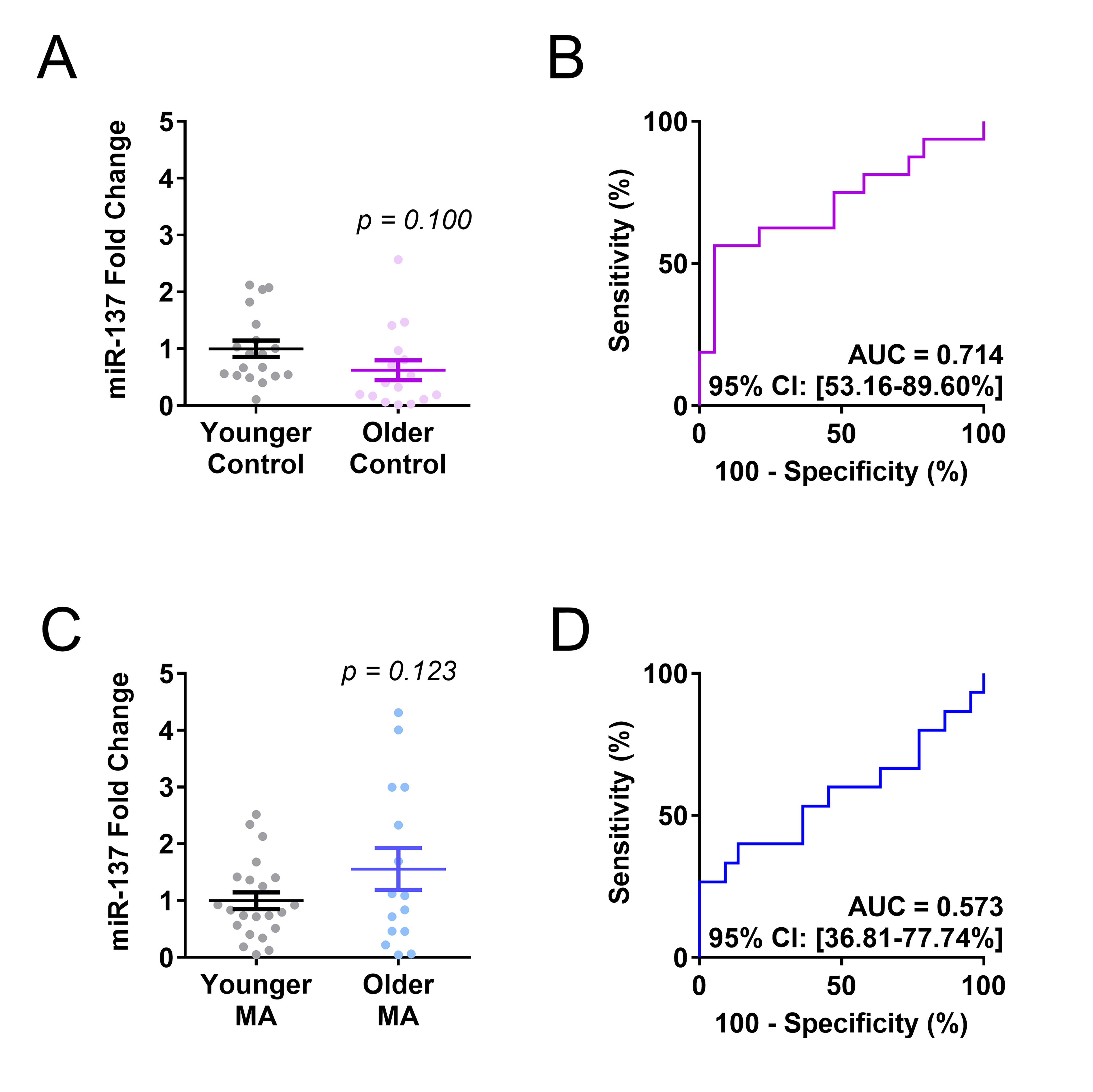

Supplement: Supplementary file 1 — (PNG 935 kb) [file 213_2022_6074_Fig5_ESM.png]

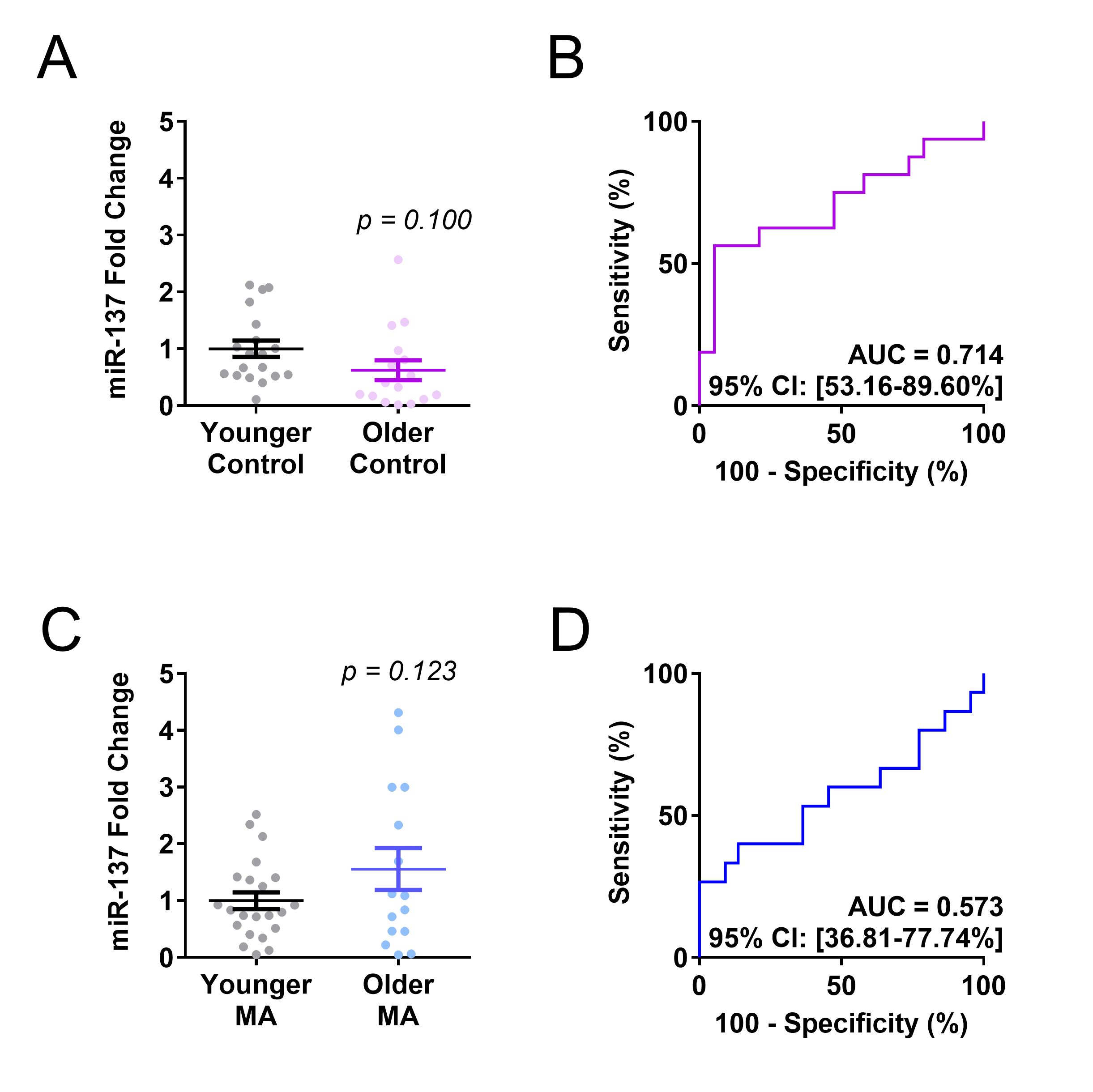

Supplement: Supplementary file 2 — High resolution image (TIF 656 kb) [file 213_2022_6074_MOESM1_ESM.tif]

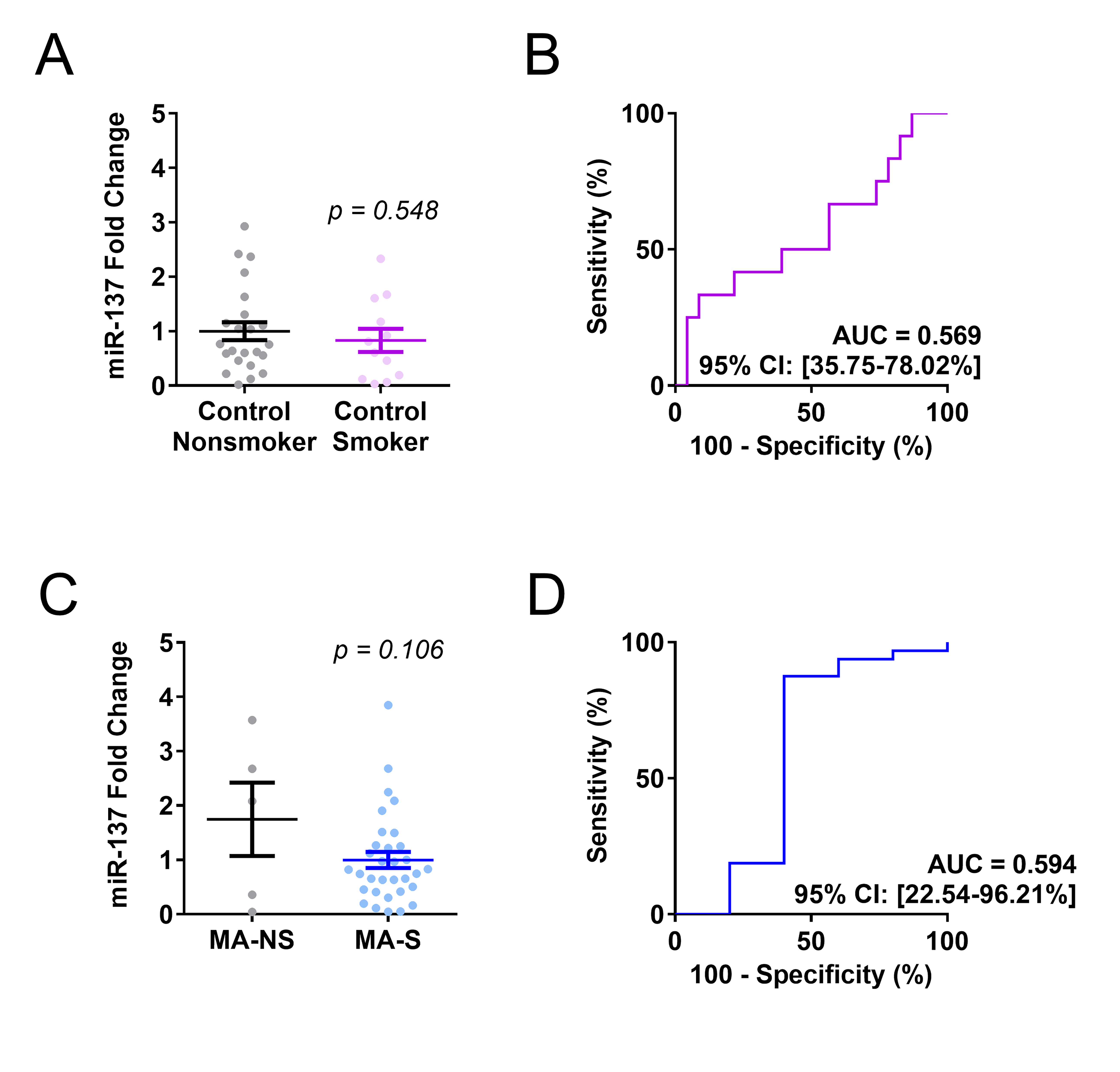

Supplement: Supplementary file 3 — (PNG 952 kb) [file 213_2022_6074_Fig6_ESM.png]

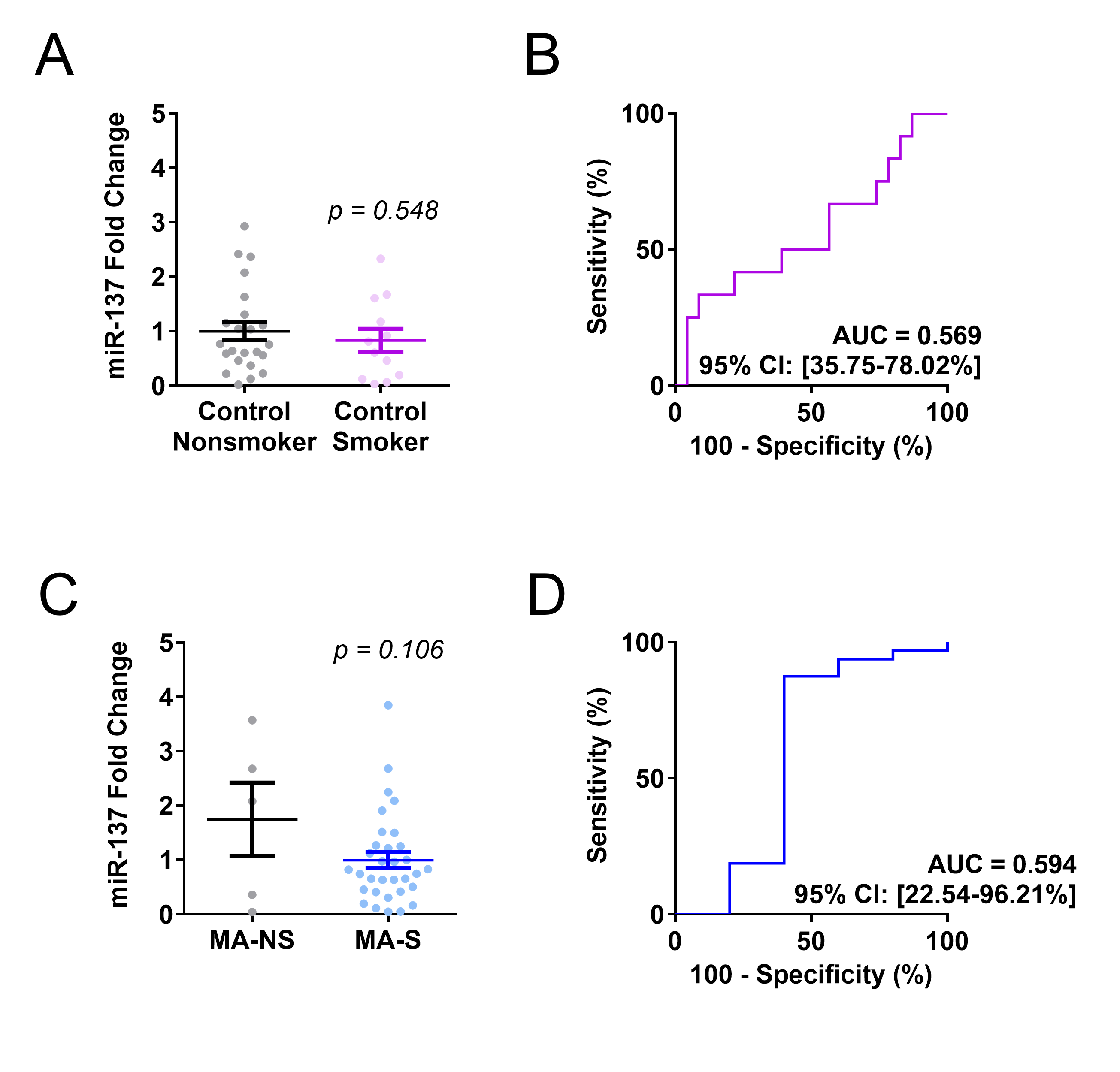

Supplement: Supplementary file 4 — High resolution image (TIF 681 kb) [file 213_2022_6074_MOESM2_ESM.tif]
